# Ethylene Signaling Facilitates Plant Adaption to Physical Barriers

**DOI:** 10.3389/fpls.2021.697988

**Published:** 2021-07-29

**Authors:** Simu Liu, Hui Chen

**Affiliations:** ^1^Guangdong Provincial Key Laboratory for Plant Epigenetics, College of Life Sciences and Oceanography, Shenzhen University, Shenzhen, China; ^2^Guangdong Key Laboratory of Genome Instability and Human Disease, School of Medicine, Shenzhen University, Shenzhen, China

**Keywords:** ethylene signaling, mechanical impedance, morphological changes, seedling emergence, root elongation, parasitic plant invasion

## Abstract

The morphological changes are usually observed in the terrestrial plants to respond to physical barriers. The phytohormone ethylene plays an essential role in the morphological development of plants encountering exogenous mechanical impedance, which enables plants to grow optimally in response to physical barriers. Ethylene is shown to regulate these developmental processes directly or in concert with other phytohormones, especially auxin. In this mini review, the involvement of ethylene action in seedling emergence from the soil, root movement within the soil, and parasitic plant invasion of the host plant are described.

## Introduction

In natural environments, the life cycle of terrestrial plants usually starts with the germination of seeds buried under the soil. After germination, seedlings need to successfully fulfill emergence from the soil to establish a photoautotrophic lifestyle, and the roots of seedling should forage soil for mineral nutrients and water acquisition. During these processes, the shoots and roots are subjected to mechanical stress from the soil. In addition, parasitic plants, whose survival depends on the host plants, encounter the physical barrier provided by the host plants, thereby they need to penetrate through the host tissues to access nutrients. To acclimate to these physical barriers, plants adopt a developmental strategy by which they adjust their morphological modifications in accordance with the mechanical impedance (Leivar et al., 2008; Yoshida et al., 2016; Gommers and Monte, 2018; Vaseva et al., 2018), such as hypocotyl thickening, apical hook formation, and root shortening occur within autotrophic plants and haustoria emergence within parasitic plants.

It has been reported that mechanical impedance boosts the production of ethylene (Goeschl et al., 1966; Zhong et al., 2014). As a gaseous plant hormone, ethylene diffuses rapidly and regulates diverse developmental and physiological processes in plants such as seedling emergence, root elongation, leaf senescence, fruit ripening, root nodulation, as well as biotic and abiotic stress responses (Bleecker and Kende, 2000). It is not surprising that terrestrial plants employ ethylene as the vital signal molecule to regulate the directional growth of plant organs since the ethylene signaling system existed prior to the plant colonization of land (Ju et al., 2015). In this mini review, we aimed to summarize current advances regarding ethylene regulation of morphological changes in facilitating autotrophic plant adaption to soil-provided physical barriers, as well as parasitic plant conquest to host plant-created physical barriers.

## Ethylene Signaling Pathway

In 1901, Dimitry Neljubov described the effects of ethylene on the growth of etiolated pea seedlings, a morphology that by 1913 was termed as the “triple response” in some dicots showing shorter and thicker hypocotyl, an exaggerated apical hook, and a shorter and swelling root (Bakshi et al., 2015). This response was utilized to genetically screen the mutants with altered ethylene response in dicotyledonous *Arabidopsis thaliana* (Bleecker et al., 1988; Guzmán and Ecker, 1990). The main components of ethylene signaling have been identified, including five endoplasmic reticulum (ER)-localized receptors, namely, ethylene response 1 (ETR1), ethylene response sensor 1 (ERS1), ETR2, ERS2, and ethylene insensitive 4 (EIN4), the negative regulator, namely, constitutive triple response 1 (CTR1), the central mediator EIN2, and the master transcription factors, namely, ethylene insensitive 3 (EIN3) and EIN3-like 1 (EIL1).

**FIGURE 1:**
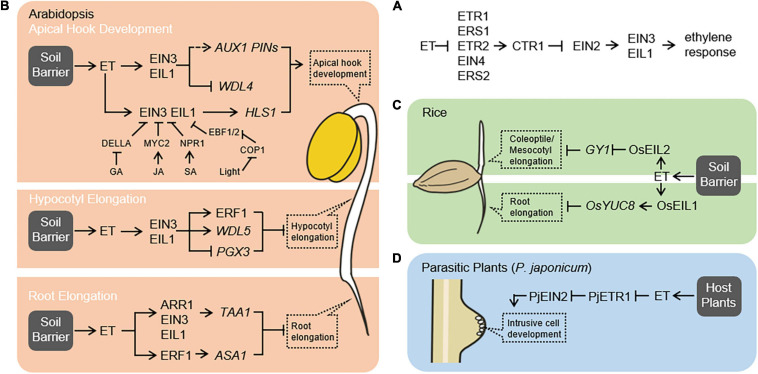
Ethylene signaling regulates the growth and development of plants encountering physical barriers. **(A)** The linear model of the ethylene signaling pathway in *Arabidopsis*. Ethylene signaling involves ethylene receptors [ethylene response 1 (ETR1), ethylene response sensor 1 (ERS1), ETR2, ethylene insensitive 4 (EIN4), and ERS2], the negative regulator [constitutive triple response 1 (CTR1)], the central mediator (EIN2), and the master transcription factors [EIN3/EIN3-like 1 (EIL1)]. The linear ethylene signaling pathway is conserved between rice and *Arabidopsis* (Yang et al., 2015a). **(B)** Ethylene facilitates *Arabidopsis* adaption to soil barriers. Ethylene regulates apical hook development by two different pathways. On the one hand, ethylene reinforces asymmetric auxin distribution within the apical hypocotyl by inducing the expression of *AUX1* and *PIN*s, and by repressing the expression of *WDL4* that influences PIN protein trafficking (Vandenbussche et al., 2010; Zádníková et al., 2010; Deng et al., 2021). On the other hand, ethylene regulates differential growth of apical hypocotyl through EIN3/EIL1 transcriptional regulation of *HLS1* expression (Shen et al., 2016). Other endogenous phytohormones including gibberellic acid, jasmonic acid, and salicylic acid and light signal also modulate ethylene-regulated apical hook development by influencing the EIN3/EIL1-*HLS1* module (An et al., 2012; Zhang et al., 2014; Shi et al., 2016a; Huang et al., 2020). Ethylene inhibits hypocotyl elongation by activation of ERF1 pathway, upregulation of *WDL5* expression, and downregulation of *PGX3* expression *via* EIN3-dependent signaling pathway (Zhong et al., 2014; Sun et al., 2015; Wu et al., 2020). Ethylene inhibits root elongation by induction of auxin biosynthesis through upregulating *WEI8/TAA1* and *ASA1* expression in EIN3- and ARR1-dependent and ERF1-dependent manner, respectively (Stepanova et al., 2005, 2008; Mao et al., 2016; Yan et al., 2017). **(C)** Ethylene facilitates rice adaption to soil barriers. In monocot rice seedlings, ethylene promotes both coleoptile and mesocotyl elongation by repression of *GY1* expression *via* OsEIL2-dependent signaling pathway (Xiong et al., 2017). Ethylene inhibits root growth by promoting auxin biosynthesis through inducing *OsYUC8* expression in an OsEIL1-dependent manner (Qin et al., 2017). **(D)** Ethylene signaling is required for the parasitic plant to invade the host plant. In the parasitic plant *Phtheirospermum japonicum*, the PjETR1 and PjEIN2, homologs of *Arabidopsis* ETR1 and EIN2, are required for intrusive cell development in response to ethylene produced by the host plants (Cui et al., 2020).

The ethylene signaling pathway in plants is well characterized (Figure 1A). In the absence of ethylene, the active receptors recruit CTR1 to phosphorylate EIN2 and thus lead to EIN2 degradation mediated by the 26S proteasome, which represses the downstream signaling pathway. In the presence of ethylene, the receptors are inactivated, reducing CTR1-mediated EIN2 phosphorylation and consequently resulting in EIN2 C-terminal end (EIN2-C) cleavage from ER-bound EIN2-N portion (Ju et al., 2012; Qiao et al., 2012). The EIN2-C enters the cytoplasmic P-body, where it represses the translation of EIN3-degrading factors, namely, EIN3-binding F-box proteins 1 and 2 (EBF1/2) and stabilizes EIN3/EIL1 proteins (Li et al., 2015; Merchante et al., 2015). EIN2-C is also able to translocate from the cytoplasm to the nucleus, where it associates with a histone binding protein for epigenetically facilitating EIN3/EIL1-dependent transcriptional regulation (Zhang et al., 2017). EIN3/EIL1 can function as a transcriptional activator or repressor in response to ethylene (Wang et al., 2020).

Although the linear ethylene signaling pathway is conserved between monocotyledonous rice and dicotyledonous *Arabidopsis* (Yang et al., 2015a), the exogenous application of ethylene inhibits root growth but promotes coleoptile growth of etiolated rice seedlings (Ma et al., 2013). Different from *Arabidopsis* EIN3 and EIL1, which regulate both shoot and root responses to ethylene, the rice EIN3 transcription factors, namely, OsEIL1 and OsEIL2, mediate the inhibition of root elongation and promotion of coleoptile elongation, respectively (Yang et al., 2015b). Unlike rice, ethylene inhibits coleoptile elongation in other monocots, including maize, wheat, sorghum, and *Brachypodium distachyon* (Yang et al., 2015a). Considering that rice is cultivated mainly in the frequently flooded river deltas, ethylene-promoted tissue elongation *via* gibberellin may contribute to diminish flooding stress (Hattori et al., 2009). These findings suggest a pivotal role of ethylene in modulating plant developmental processes to acclimate to ever-changing environments.

## Ethylene Signaling Is Required for Shoots to Emerge From the Soil

To overcome the soil barrier and reach sunlight, seedlings frequently adopt a developmental strategy dynamically adjusting their morphological structures in accordance to soil depth (Zhong et al., 2014). In *Arabidopsis*, the short and thick hypocotyl is suitable for enhancing the lifting capacity of etiolated seedlings, and the apical hook is an elegant structure for protecting the delicate shoot apical meristem against the mechanical damage when seedlings emerge from the soil. In rice, the coleoptile and mesocotyl are the two essential structures of seedlings responsible for moving toward the soil surface (Lee et al., 2017; Xiong et al., 2017). The coleoptile, which wraps the emergent shoot, safeguards the plumule against mechanical injures. The mesocotyl, a structure between the coleoptile node and the basal part of the seminal root in etiolated monocot seedlings, pushes the buds out of the soil. It has been reported that the soil-imposed mechanical impedance stimulates the production of ethylene (Goeschl et al., 1966; Zhong et al., 2014; Xiong et al., 2017), which acts as a key signal linking plant responses to the environmental cues (Sarquis et al., 1991; He et al., 1996).

## Ethylene Suppresses Hypocotyl Elongation in Response to Soil Barrier

In *Arabidopsis*, the subterranean hypocotyl growth during soil emergence is an important phase for seedling establishment and survival. Under the soil, ethylene employs diverse pathways to suppress the hypocotyl elongation and increase the hypocotyl strength (Figure 1B), hence improves the capacity of hypocotyl response to soil barrier. Ethylene activates the ethylene response factor 1 (ERF1) pathway in an EIN3-dependent manner to slow down hypocotyl elongation and thus enables seedlings to penetrate through the soil layers more easily (Zhong et al., 2014). Ethylene is also able to inhibit hypocotyl cell elongation by upregulating *wave-dampened2-like 5* (*WDL5*) gene expression through the EIN3-dependent signaling pathway (Sun et al., 2015). *WDL5* encodes a microtubule-associated protein that stabilizes the microtubule of cortical cells in the etiolated hypocotyl. In addition, ethylene suppresses pectin degradation and thus enhances cell wall stiffness by EIN3-mediated downregulation of *polygalacturonase involved in expansion* 3 (*PGX3*) gene expression (Wu et al., 2020). This EIN3-*PGX3* regulatory module contributes to the inhibition of hypocotyl elongation as well as the facilitation of seedling soil emergence. Although ethylene inhibition of hypocotyl elongation may delay seedlings reaching the sunlight, a longer period of etiolated growth is beneficial for EIN3-phytochrome interacting factor 3 (PIF3)-modulated preassembly of photosynthetic machinery, which is necessary for the seedling transition from dark to light (Zhong et al., 2012, 2014).

## Ethylene Regulates Apical Hook Development During Soil Emergence

The differential distribution of auxin across the apical hypocotyl region and accumulation on the concave (inner) is essential for regulating asymmetric cell elongation between concave and convex (outer) and consequently leading to apical hook formation (Raz and Ecker, 1999; Béziat and Kleine-Vehn, 2018). Auxin distribution is facilitated by the auxin influx carrier auxin-resistant 1 (AUX1) and the efflux carrier pin-formed (PIN) family proteins (Petrásek and Friml, 2009). During the early phases of seedling germination, a gravity-stimulated and PIN2-directed asymmetric auxin distribution in the root is able to extend into hypocotyl and thus initiates the establishment of asymmetric auxin distribution in this zone (Zhu et al., 2019). Ethylene-induced expression of *AUX1* and *PINs* within the apical hypocotyl may further reinforce this initial auxin asymmetry (Vandenbussche et al., 2010; Zádníková et al., 2010). Moreover, ethylene represses the expression of *WDL4 via* the EIN3-dependent signaling pathway, which influences PIN protein trafficking and auxin asymmetric distribution (Figure 1B; Deng et al., 2021). Then, the transmembrane kinase 1 (TMK1)-mediated auxin response pathway translates differential cellular auxin to differential cell growth during apical hook formation (Cao et al., 2019). A recent finding also supports that PIN-facilitated polar auxin transport followed by TMK-mediated auxin signaling pathway is required for mechanically induced apical hook formation (Baral et al., 2021).

Besides depending on the auxin pathway, ethylene regulates apical hook development through EIN3/EIL1 transcriptional regulation of *hookless* 1 (*HLS1*) expression (EIN3/EIL1-*HLS* module) (Figure 1B; Shen et al., 2016). *HLS1* encodes a putative *N*-acetyltransferase, and the loss of function of the *HLS1* gene completely disrupts apical hook curvature (Lehman et al., 1996), indicating an essential role of HLS1 in apical hook formation. Although the biochemical nature of HLS1 protein remains mysterious, the formation of HLS1 oligomer is required for its function during apical hook formation (Lyu et al., 2019). Other endogenous phytohormones and light signal also modulate apical hook development by influencing the EIN3/EIL1-*HLS1* module. In darkness, the gibberellin-repressed DELLA, jasmonic acid-activated MYC2, or salicylic acid-activated NPR1 can directly interact with and alter the transcriptional activity of EIN3/EIL1 (An et al., 2012; Zhang et al., 2014; Huang et al., 2020). The photomorphogenic central repressor constitutive photomorphogensis 1 (COP1) is also able to stabilize EIN3/EIL1 protein by directly ubiquitinating EBF1/2 for degradation (Shi et al., 2016a). It is still unknown how HLS1 regulates asymmetric cell growth during apical hook formation. A potential explanation may be that HLS1 mediates ethylene-repressed accumulation of auxin response factor 2 (ARF2) protein, which is an auxin response transcription factor negatively regulating differential auxin response during apical hook formation (Li et al., 2004).

Upon seedling reaching the soil surface, the mechanical force-stimulated production of ethylene decreases, whereas the light exposure increases. The light represses COP1 nuclear localization and thus releases EBF1/2 for EIN3 degradation (Shi et al., 2016a). Meanwhile, the light activates the photoreceptor phytochrome B (PhyB) and promotes its translocation into the nucleus, where it enhances EBF1/2-stimulated EIN3/EIL1 degradation and disrupts HLS1 oligomer formation as well (Shi et al., 2016b; Lyu et al., 2019). Therefore, the light-regulated EIN3/EIL1 reduction inactivates ethylene signaling, which attenuates hook curvature but facilitates hook opening and cotyledon expansion to initiate photomorphogenic growth programs.

## Ethylene Promotes Elongation of Coleoptile and Mesocotyl in Rice

In *Arabidopsis* seedlings, thickened hypocotyl and apical hook are the key morphological structures facilitating the soil emergence, while in rice seedlings they are coleoptile and mesocotyl. Ethylene-promoted coleoptile and mesocotyl elongation are mediated by transcription factor OsEIL2 in rice seedlings (Figure 1C). OsEIL2 directly represses transcription of the *gaoyao 1 (GY1)*, which encodes a phospholipase that functions at the initial step of jasmonic acid biosynthesis (Xiong et al., 2017). Consistent with this, ethylene emission increases while jasmonic acid content decreases in the rice seedlings with increasing seed-sowing depth of soil, suggesting that ethylene stimulates coleoptile and mesocotyl elongation to facilitate seedling emergence by inhibition of jasmonic acid biosynthesis (Xiong et al., 2017). Overexpression of *OsEIN2* also leads to a longer coleoptile and longer mesocotyl of etiolated rice seedlings in the presence of ethylene (Ma et al., 2013), indicating a major role of ethylene in promoting coleoptile and mesocotyl elongation in rice seedlings. Other phytohormones such as brassinosteroids and strigolactones are also involved in regulating mesocotyl growth (Sun et al., 2018; Zheng et al., 2020). How ethylene coordinately with other hormones modulates mesocotyl elongation during rice seedling emergence from the soil needs more detailed studies.

## Ethylene Signaling Is Required for Root Adaption to Soil Barrier

In most cases, the shoots encounter soil barriers only before they emerge from the soil, whereas the roots have to face persistent soil barriers in the whole life cycle. Thus, the roots need to continuously adjust their morphological structures to respond to ever-changing local soil environments. The physically impeded roots display diverse morphological alterations, such as inhibition of root elongation and increase of radial dimension, resembling the morphological changes observed when roots are grown in the presence of ethylene (Jacobsen et al., 2021). Consistent with these observations, when ethylene biosynthesis or signaling is repressed, the degree of root response to mechanical impedance is significantly alleviated (Santisree et al., 2011). Interestingly, the loss of function of *WDL5* gene, which is a target of EIN3, impairs mechanical stress-inhibited elongation of both hypocotyl and root (Sun et al., 2015; Okamoto et al., 2021), suggesting that a general mechanism regulating cell elongation should be crucial for ethylene regulation of morphological changes in response to mechanical impedance.

The root cap, which is located at the apex of the root and protects the root apical meristem, is able to sense mechanical stimulus and control the direction of root growth (Dreyer and Edelmann, 2018). Removal of root cap disturbs ethylene-inhibited root growth and affects root exploration and penetration in the soils, indicating that the root cap should be a potential site of the ethylene-regulated root–soil interaction (Hahn et al., 2008). When in contact with the soil, the root cap needs to evaluate the resistance strength of the soil and then takes the decision to penetrate through the soil layers or avoidance of obstacles. Charles and Francis Darwin have described this intelligent root cap in their book “*The Power of Movement of Plants*” (Darwin and Darwin, 1881). Ethylene emission and accumulation around the root tip tissues may be crucial for root caps to choose the above two different strategies according to the soil conditions. Ethylene-promoted increased diameter of roots may lead to favorable mechanical strength to penetrate through the penetrable soil layers; alternatively, ethylene-inhibited root elongation may facilitate avoidance of compacted soils. In agreement with this view, compacted soil-restricted ethylene diffusion triggers a rapid and sustained increase of EIN3-green fluorescent protein in cell nuclei at the root elongation zone, indicating that ethylene signaling is strongly activated in this zone. Consistently, the roots of *Osein2* and *Oseil1* mutants that are insensitive to ethylene penetrate compacted soil more effectively than wild-type’s roots (Pandey et al., 2021).

In screening for mutants that display ethylene insensitivity in roots of *Arabidopsis*, mutants related to auxin biosynthesis, auxin transport, and auxin signaling were identified (Stepanova et al., 2007). In *Arabidopsis*, the main auxin is indole-3-acetic acid (IAA), which is mainly synthesized by the indole-3-pyruvic acid (IPA) pathway using tryptophan (Trp) as a precursor (Zhao, 2012). Upon perception of auxin, transport inhibitor response 1 (TIR1) and auxin signaling F-box proteins (AFBs) physically interact with auxin or IAA (Aux/IAA) transcriptional repressors and promote the degradation of them, which release ARF transcription factors to activate diverse auxin-responsive genes (Chapman and Estelle, 2009).

The *weak ethylene insensitive 2/anthranilate synthase alpha 1* (*WEI2*/*ASA1*) and *WEI7/anthranilate synthase beta 1* (*ASB1*) genes encode rate-limiting enzyme of Trp biosynthesis. Ethylene inhibits root elongation by upregulation of WEI2/ASA1 and WEI7/ASB1 to lead to auxin accumulation in the root tip, and ERF1 is shown to physically bind to the *ASA1* promoter and induces its expression (Stepanova et al., 2005; Mao et al., 2016). The *WEI8*/*tryptophan aminotransferase of Arabidopsis 1* (*TAA1*) and *Yucca* (*YUC*) encode two key enzymes in the IPA pathway and catalyze the conversion of Trp to IPA and IPA to IAA, respectively (Zhao, 2012). EIN3 regulates *WEI8*/*TAA1* expression by interacting with and enhancing response regulator 1 (ARR1) transcriptional activity (Figure 1B; Stepanova et al., 2008; Yan et al., 2017). In rice, OsEIL1 is able to directly target the *OsYUC8* promoter to regulate its expression (Figure 1C; Qin et al., 2017). In addition, reduced expression of rice auxin receptor genes *OsTIR1* and *OsAFB2* cause root ethylene insensitive (Chen et al., 2018). Furthermore, loss of function of *soil-surface rooting 1* (*SOR1*), an E3 ubiquitin ligase modulating protein stability of OsIAA26 and OsIAA9, also leads to defective of root ethylene response (Chen et al., 2018). A recent finding shows that auxin is involved in root-obstacle avoidance and especially the PIN-mediated polar auxin transport facilitates this process (Lee et al., 2020). These findings indicate that ethylene largely employs the auxin pathway to regulate root growth.

## Ethylene Signaling Is Required for the Parasitic Plant to Invade the Host Plant

Unlike most of plants whose shoots and roots mainly encounter soil-caused physical barriers, parasitic plants majorly experience another type of physical barrier organized by the host plants (Twyford, 2018). The parasitic plants need to obtain all or part of nutrients from host plants to support their survival, so they form a special morphological structure termed haustoria to attach to hosts, penetrate through the host tissues, and ultimately establish the physiological conduit (Clarke et al., 2019). The *Orobanchaceae* parasitic plants are root parasitic plants that establish a connection with host plants by the formation of haustorium. The *Triphysaria versicolor* and *Phtheirospermum japonicum* belong to *Orobanchaceae* have been studied in the laboratory to expand our understanding of communication between parasitic and host plants.

After germination, *Orobanchaceae* parasitic plants initiate haustorium formation since they recognize active haustorium-inducing factors (HIFs) such as 2,6-dimethoxy-*p*-benzoquinone (DMBQ), produced by the potential hosts. Upon contact to host roots, a haustorium initiates invasion using the intrusive cells potentially differentiated from epidermal cells at the apex. In *T. versicolor*, the production of ethylene increases in response to DMBQ, and the blockage of ethylene biosynthesis or signaling almost completely suppresses haustorium formation, suggesting that ethylene is involved in haustorium development (Tomilov et al., 2005). To uncover the genetic programs of haustorium development, a genetic screening was performed using *P. japonicum*, and the mutation of *PjETR1* and *PjEIN2*, encoding a homolog of *Arabidopsis ETR1* and *EIN2*, respectively, were identified (Figure 1D). Both mutants displayed elongated haustorium but failed host invasion. Further observations suggest that parasitic plants initiate haustorium development when they detect HIFs and maintain haustorium elongation until they sense host-derived ethylene, which inhibits haustorium elongation but promotes differentiation of haustorium apex cells into intrusive cells for host invasion (Cui et al., 2020). These findings indicate that ethylene signaling is essential for parasitic plants to overcome physical barriers provided by host plants.

## Conclusion and Perspectives

As a plant hormone, ethylene promotes the environmental adaption of plants by regulating diverse morphological development. In this mini review, we described the role of ethylene in facilitating plant adaption to diverse physical barriers for survival. To establish a photoautotrophic lifestyle, ethylene promotes hypocotyl shortening and apical hook formation in *Arabidopsis* and both coleoptile and mesocotyl elongation in rice to support them to move through the soils more effectively and safely. To uptake water and mineral nutrients from the soils, ethylene modulates root growth for optimal response to soil barriers. To establish a successful parasitic relationship, ethylene promotes host invasion by stimulating intrusive cell development at the haustorium apex.

Although some targets regulated directly by EIN3/EIL1 have been identified (Figure 1), it remains largely unclear how mechanical impedance triggers ethylene action. Mechanosensitive ion channels are a common mechanism for sensing mechanical impedance. In *Arabidopsis*, the MID1-complementing activity 1 (MCA1) is a Ca^2+^-permeable mechanosensitive channel enabling roots to overcome mechanical barriers (Okamoto et al., 2021), and the piezo1 (PZO1) is another ion channel that affects Ca^2+^ transients in response to mechanical stimulation and is required for roots to penetrate hard agar (Mousavi et al., 2021). The ethylene is biosynthesized by two dedicated enzymatic reactions. The substrate *S*-adenosyl-L-methionine is converted to the ethylene precursor 1-aminocyclopropane-1-carboxylic acid (ACC) by the ACC synthase (ACS), and then the ACC is converted to ethylene by the ACC oxidase (ACO) (Pattyn et al., 2021). In *Vigna radiata*, the AIM-1 is a key ACS, whose mRNA levels dramatically increase in the presence of mechanical stimulation (Botella et al., 1995). In *Arabidopsis*, the *ACS6* expression was induced by touch (Arteca and Arteca, 1999). It will be interesting to study whether and how mechano-sensitive ion channel-mediated increase of cytoplasmic Ca^2+^ upregulates *ACS* gene expression and thus triggers ethylene production and signaling.

Characterizing the role of ethylene in facilitating plant adaption to physical barriers at the levels of molecular biology, biochemistry, and cellular biology is therefore important not only to expand our understanding of the plant–soil/plant–plant interaction but also to breed crops that can grow optimally in unfavorable lands with compact soils and develop a useful strategy to control parasitic plant-caused cereal yield losses.

## Author Contributions

Both authors listed have made a substantial, direct and intellectual contribution to the work, and approved it for publication.

## Conflict of Interest

The authors declare that the research was conducted in the absence of any commercial or financial relationships that could be construed as a potential conflict of interest.

## Publisher’s Note

All claims expressed in this article are solely those of the authors and do not necessarily represent those of their affiliated organizations, or those of the publisher, the editors and the reviewers. Any product that may be evaluated in this article, or claim that may be made by its manufacturer, is not guaranteed or endorsed by the publisher.
